# Nature Inspired MXene-Decorated 3D Honeycomb-Fabric Architectures Toward Efficient Water Desalination and Salt Harvesting

**DOI:** 10.1007/s40820-021-00748-7

**Published:** 2021-12-04

**Authors:** Zhiwei Lei, Xuantong Sun, Shifeng Zhu, Kai Dong, Xuqing Liu, Lili Wang, Xiansheng Zhang, Lijun Qu, Xueji Zhang

**Affiliations:** 1 College of Textiles and Clothing, State Key Laboratory of Bio-Fibers and Eco-Textiles, Research Center for Intelligent and Wearable Technology, Intelligent Wearable Engineering Research Center of Qingdao, Qingdao University, Qingdao, 266071 People’s Republic of China; 2 Department of Materials, University of Manchester, Manchester, M13 9PL UK; 3 Beijing Institute of Nanoenergy and Nanosystems, Chinese Academy of Sciences, Beijing, 100083 People’s Republic of China; 4 School of Biomedical Engineering, Health Science Center, Shenzhen University, Shenzhen, 518060 People’s Republic of China

**Keywords:** 3D honeycomb fabric, MXene, Photothermal conversion, Water desalination, Salt harvesting

## Abstract

- The 3D honeycomb-like fabric decorated with MXene is woven as solar evaporator.
- The honeycomb structure enables light-trapping and recycling of convective and radiative heat.
- The 3D honeycomb-fabric evaporator possesses high solar efficiency up to 93.5% under 1 sun irradiation and excellent salt harvesting ability.

**Abstract:**

Solar steam generation technology has emerged as a promising approach for seawater desalination, wastewater purification, etc. However, simultaneously achieving superior light absorption, thermal management, and salt harvesting in an evaporator remains challenging. Here, inspired by nature, a 3D honeycomb-like fabric decorated with hydrophilic Ti_3_C_2_T_x_ (MXene) is innovatively designed and successfully woven as solar evaporator. The honeycomb structure with periodically concave arrays creates the maximum level of light-trapping by multiple scattering and omnidirectional light absorption, synergistically cooperating with light absorbance of MXene. The minimum thermal loss is available by constructing the localized photothermal generation, contributed by a thermal-insulating barrier connected with 1D water path, and the concave structure of efficiently recycling convective and radiative heat loss. The evaporator demonstrates high solar efficiency of up to 93.5% and evaporation rate of 1.62 kg m^−2^ h^−1^ under one sun irradiation. Moreover, assisted by a 1D water path in the center, the salt solution transporting in the evaporator generates a radial concentration gradient from the center to the edge so that the salt is crystallized at the edge even in 21% brine, enabling the complete separation of water/solute and efficient salt harvesting. This research provides a large-scale manufacturing route of high-performance solar steam generator.
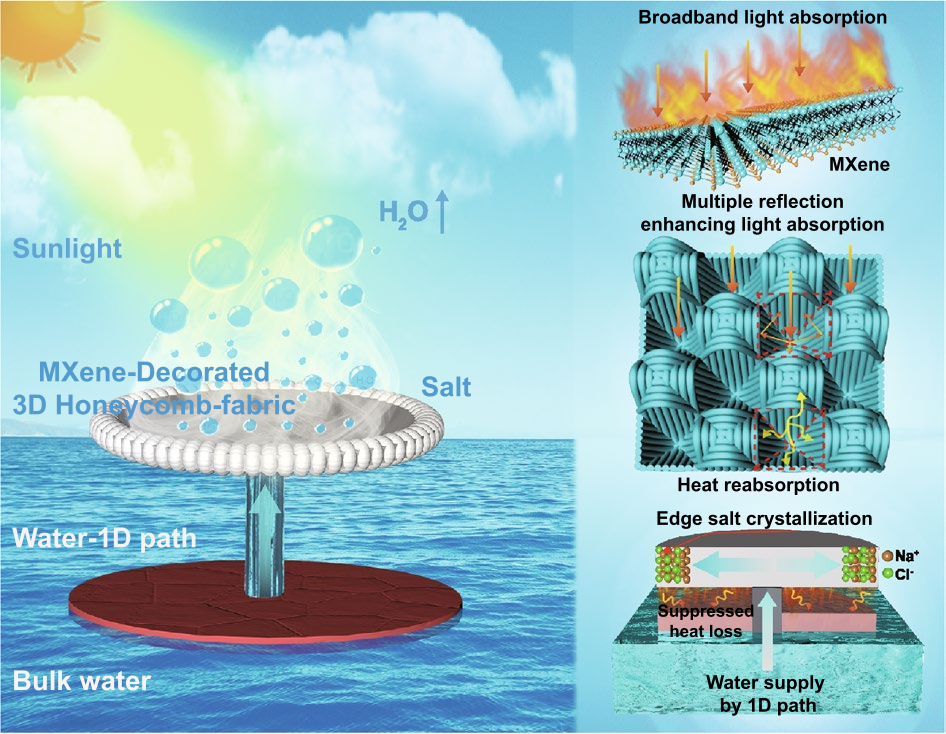

**Supplementary Information:**

The online version contains supplementary material available at 10.1007/s40820-021-00748-7.

## Introduction

Nowadays, the conflict between population growth and freshwater shortage has become one of the most challenging issues in the twenty-first century [1]. Estimates indicate that the number of persons living in water-scarce areas may increase to 3.9 billion by 2025 [2]. Therefore, efficient methods to develop freshwater resources have become an important research topic [3]. Solar energy, as the largest renewable and sustainable energy resource available, presents various applications in desalination, wastewater purification, and large-scale power generation, which are driven by interfacial vapor evaporation via solar-thermal technology [4–7]. Efficient absorption of broad-spectrum solar radiation and excellent thermal management could remarkably improve the efficiency of solar-driven interfacial evaporation systems and provide a promising means to solve the shortage of freshwater resources [8, 9].

An ideal solar steam generation system should be capable of efficient solar energy absorption and high solar-thermal conversion efficiency; it must also possess excellent thermal management performance, efficient water transfer capability, superior salt resistance, long-term stability, and scalable production [10–14]. Researchers have proposed the following several approaches to optimize the different functional components of solar steam generation evaporators. (1) Explorations of photothermal convention materials, such as semiconductors [15], metal nanoparticles [16], carbon-based materials [17, 18], and conjugated polymers [19], have been attempted. However, low energy-conversion efficiency remains a common issue in the developed solar absorbers. (2) In term of the absorber structure, the theoretical limit of energy efficiency of traditional state-of-the-art evaporators with two-dimensional (2D) planar structures has been reached. Planar structures generally lead to high light reflection, thermal radiation, and thermal convection, resulting in large energy loss [20]. Several researchers have sought to synthesize three-dimensional (3D) macrostructures [21], such as origami 3D tessellation, conical shape, lotus flowers, origami rose, and cylindrical cup-shaped structures, to enable incident light to undergo multiple reflections and minimize energy loss, thereby contributing to the light absorption and evaporation efficiency of the evaporators [20, 22–25]. (3) In the case of water transport channels, 3D vessels with random and interconnected porous structures, such as aerogels with vertically aligned vessels and inherent porous networks, wood with interconnected micro/nanochannels, and loofah fibers with hierarchical macropores and microchannels, have been adopted to achieve water transport via capillary effects [26–32]. However, water in the transfer channels could increase thermal conductivity and heat loss. An integrated device combining porous heat insulation foam and confined water path could overcome this problem. Water-absorbing cellulose materials have been used to provide confined 2D surface water paths; these materials are wrapped on the surface of insulation foam to guarantee the water supply and suppress heat loss [33, 34]. However, during steam generation, the crystallization of salts on the surface of photothermal materials leads to a gradual decline in the water evaporation rate. (4) During practical seawater desalination, water evaporation leads to salt accumulation on the evaporator surface, which not only seriously affects the light absorption area of solar absorbers but also inevitably blocks water transport vessels. Many scholars have designed a series of structures to avoid the accumulation of salt; these structures include drilled channels of millimeter-scale holes with high hydraulic conductivity to dissolve salt back into the bulk solution, Janus structures to make salt ions stay in the hydrophilic layer without crystallizing on the evaporator surface, and nanofiber-based evaporators to induce salt transfer from the evaporating interface to the water along the holes between nanofibers [26, 35–38]. However, heat loss continues to occur in these devices because of rapid convection, or a portion of the light inevitably passes through the holes of the evaporator. In addition, the highly concentrated salt cannot be harvested, which wastes valuable mineral resources. In summary, although recent developments in solar evaporators have resulted in a series of achievements in optimizing local structures, the defects remaining in these structures must be addressed to further enhance the photothermal conversion efficiency of the resulting devices. More importantly, the light-absorbing material, substrate structure, thermal management, water transfer, and salt barrier should be simultaneously optimized to enable the large-scale fabrication of highly efficient and stable solar evaporators. Currently, however, these endeavors continue to challenge experts.

**Fig. 1:**
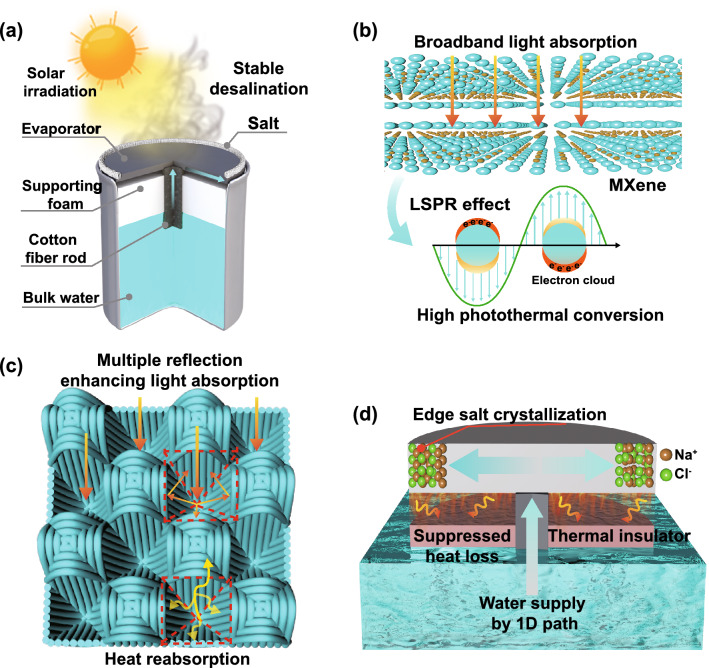
**a** Schematic diagram of MXene-decorated 3D honeycomb fabric-based solar evaporator for desalination. **b** LSPR effect-based photothermal conversion mechanism of MXene. **c** Schematic illustration of the 3D honeycomb-structured fabric for light absorption and heat reabsorption. **d** Schematic diagram of polystyrene foam (for insulation) and cotton fiber rods (for water transport) used to suppress heat loss, salt ring accumulation and 1D water pathway

In this research, inspired by nature, we innovatively designed an efficient solar vapor generation device by combining a 3D honeycomb-structured fabric (i.e., millimeter-scale honeycombed pore structure arrays) with a new photothermal conversion material (i.e., Ti_3_C_2_T_x_ MXene) (Fig. 1a). First, MXene presents high near-infrared (NIR) light absorption owing to its localized surface plasmon resonance (LSPR) effect (Fig. 1b), as well as 100% internal photothermal conversion efficiency, which facilitates the effective generation of solar steam [39–41]. Moreover, the large number of hydrophilic end groups existing on the surface of MXene is conducive to the penetration and transport of water molecules [42]. Second, the 3D honeycomb-structured fabric is characterized by a periodically concave array structure, rough surface, and high porosity (Fig. 1c). These features promote multiple scattering and omnidirectional light harvesting absorption of internal light, decreasing the thermal conductivity (< 0.05 W m^−1^ K^−1^) of the fabric and efficiently recycle radiative and convective heat losses. The ratio of the surface area to the projected area of the 3D honeycomb-structured fabric is approximately 2.06:1, which means a large surface area is available for evaporation. With the synergistic effect of the honeycomb-structured fabric and MXene on the trapping and absorption of sunlight, the surface temperature of the dried MXene/3D honeycomb-structured fabric under 1 sun irradiation could reach 86 °C, which exceeds that of most reported photothermal materials [43, 44]. Third, the minimum thermal loss is available by creating the localized photothermal generation, contributed by a thermal-insulating supporting layer connected with confined one-dimensional (1D) water transporting path. Finally, 1D cotton fiber rods with powerful capillary pumping performance were embedded at the center of the foam to transport water through the fabric. Given the excellent hydrophilic characteristics of the honeycomb fabric, salt water could spread from the cotton fiber rods toward the edges of the fabric (Fig. 1d). As the evaporation of water continues under solar exposure, an increase in the gradient of the salt concentration occurs from the center to the edge of the fabric, resulting in the crystallization of the salt at the edge of the fabric rather than on the surface of the evaporator. The ingenious design of the developed device prevents the vapor channels from becoming blocked and realizes the effective collection of salt.

Hence, in this study, the optimization of light absorption structure, thermal management, water transport, and salt rejection of the device endowed the MXene/3D honeycomb-structured fabric-based evaporator with outstanding evaporation performance. Specifically, the device could achieve a light absorption rate of 96%, evaporation rate of 1.62 kg m^−2^ h^−1^ with simultaneous salt harvesting from saline water, and solar efficiency of 93.5% under the irradiation of 1 solar intensity (1 kW m^−2^). Moreover, the MXene/3D honeycomb-structured fabric possesses excellent strength, flexibility, shape adaptation and scalability, which can easily meet the portability transport and storage requirement for outdoor use. Hence, our research provides a new scope for continuous and scalable water desalination.

## Experimental Section

### Fabrication of the 3D Honeycomb Fabric

The 3D honeycomb fabric was woven on a semi-automatic weaving sample machine (SGA598, Jiangyin Tongyuan Textile Machinery Co., Ltd.) by the interweaving of warp and weft yarns (Fig. S3). First, the design of honeycomb fabric structure was input into the control panel of the loom. Second, the warp yarns were arranged on the loom, and the warp yarns were selective separated to two parts according to requirement of the process. Third, the weft yarn was wrapped around the shuttle and the weaving process begins.

### Preparation of MXene/3D Honeycomb Fabric

The Ti_3_AlC_2_ powder was selectively etched to obtain Ti_3_C_2_T_x_ suspension according to the reported works (Fig. S1). The honeycomb fabrics were first ultrasonically cleaned with ethanol and deionized water for removing the impurities on its surface. Subsequently, the pre-cleaned fabrics were immersed in 1 mg mL^−1^ PDDA solution and soaked for 30 min, then rinsed with deionized water for 5 min to remove weakly absorbed molecules. The obtained fabrics were dried at 60 °C for 2 h. Afterward, the cation-coated fabric was immersed in MXene dispersion for 5 min and dried in a vacuum oven at 50 °C for 4 h. The dip-coating process was repeated following the above procedures. To determine the mass loading of the MXene flakes, the samples were weighed before and after the dipping process.

### Characterization

The morphology of MXene nanosheet was obtained by transmission electron microscopy (TEM, JEM-2100, JEOL, Japan) and atomic force microscopy (AFM, Agilent 5400). The size of MXene particles and the Zeta potential were measured on Zetasizer Nanoseries (NanoZS90, Malvern Instrument Ltd). The morphology and the corresponding elemental mapping of fabrics were investigated by emission scanning electron microscope (SEM, EVO18, ZEISS, Germany) with energy dispersive X-ray (EDS) spectroscopy. The morphology of the surface and honeycomb structure of single yarns were characterized using a 3D optical microscope (Keyence VHX-S50). The crystal property and composition of the fabricated samples were collected by X-ray diffractometer (XRD, SmarlabSE, Japan) with Cu Kα radiation (*λ* = 1.5406 Å). The chemical composition of the samples was characterized by X-ray photoelectron spectroscopy (XPS, Escalab 250 Xi, Thermo Scientific, America). Fourier transform infrared (FTIR) spectrometer (Thermo Scientific Nicolet iS50) was used to obtain FTIR spectra. The mechanical properties were measured using a universal tensile testing machine (Instron 5965, USA). The water contact angle of the fabric was measured using Optical contact angle (OCA 15EC). The UV–vis spectroscopy (UV-3600, Shimadzu, Japan) attached with an integrating sphere was used for the optical diffuse transmittance and reflection spectra of the fabrics measurements. The absorbance was calculated by *A* = 1 − *R* − *T* according to Kirchhoff’s law, where *R* and *T* are the reflection and transmission, respectively. The concentration of the metal ion was measured with the inductively coupled plasma-optical emission spectrometry (ICP-OES, Avio 200, PE).

### Solar Steam Generation Experiment

The performance of solar vapor generation was evaluated by dedicated equipment including a solar simulation system and a weighing system. Solar radiation was simulated using a solar simulator (CEL-PE300L-3A Xenon lamp, China) with an AM1.5G, and the power density of solar irradiation was determined using an auxiliary detector (CEL-NP2000 Optical power meter, China). Here, the optical density on the fabric samples was adjusted by controlling the power and optical distance of the solar simulator. Each sample was light irradiated for 60 min and the weight could be collected every 20 s by a computer, which was connected to an electronic balance (0.1 mg in accuracy). The temperature was measured using an IR camera. All experiments were conducted at an ambient temperature of approximately 25 °C and humidity of 60% in the laboratory.

## Results and Discussion

**Fig. 2:**
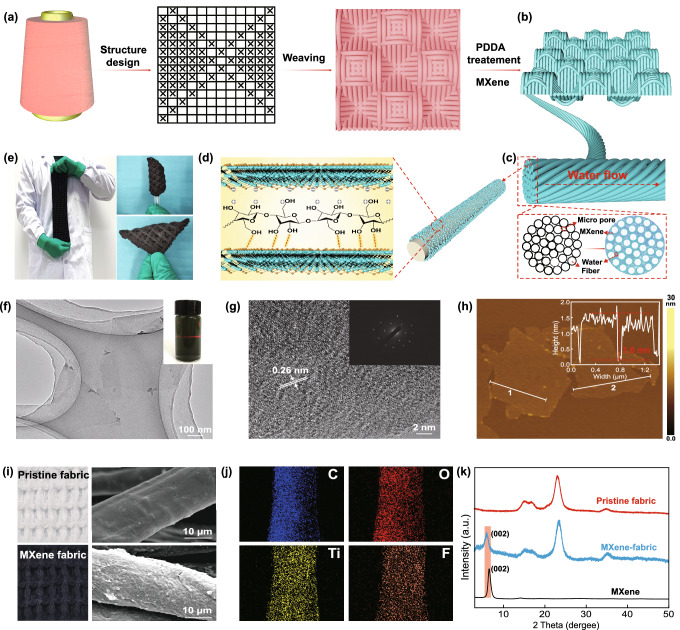
**a** Schematic diagram of the fabrication process of the 3D honeycomb fabric. **b**–**c** Schematic diagram of the 3D honeycomb fabric and yarn after PDDA/MXene coating. **d** Schematic diagram of the interactions between MXene and the fibers. **e** Digital images of the flexible MXene/3D honeycomb fabric (length, 30 cm; width, 10 cm). **f** TEM image and the Tyndall effect of MXene. **g** HRTEM image of MXene. **h** AFM image of MXene. **i** SEM images of pristine and MXene/3D honeycomb fabrics. **j** EDS elemental mapping of C, O, Ti, and F in the MXene/3D honeycomb fabric. **k** XRD mapping of MXene, the MXene/3D honeycomb and pristine 3D honeycomb fabrics

Ti_3_C_2_T_x_ (MXene) was obtained by etching the aluminum layer of Ti_3_AlC_2_ (MAX phase) with a LiF/HCl mixed solution (Fig. S1). The successful exfoliation of the MAX phase to MXene nanosheets is confirmed by XRD and XPS (Fig. S2a, b). The abundant surface terminal groups of MXene, which include –O, –OH, and –F, enable it to disperse homogeneously in water, as evidenced by its zeta potential of − 49.6 mV (Fig. S2c) and typical Tyndall effect (Fig. 2f) [45]. TEM and AFM results show that the MXene nanosheets are very thin and transparent, with a relatively uniform lateral size of approximately 300–500 nm and a thickness of approximately 1.6 nm (Figs. 2f, h and S2d). HRTEM and selected area electron diffractometry (SAED) reveal the clear lattice stripes and symmetric hexagonal structure of single crystals (Fig. 2g). These results demonstrate that high-quality and monolayer Ti_3_C_2_T_x_ nanosheets had been successfully prepared. The 3D honeycomb-structured cotton fabric features a periodic arrangement of concave honeycomb structures woven by warp and weft yarns (Figs. 2a and S3). MXene/3D honeycomb-structured fabrics were fabricated by a simple and scalable self-assembly strategy. Here, the honeycomb-structured fabrics are first treated with positively charged poly (diallyldimethylammonium chloride) (PDDA) and then impregnated in a negatively charged MXene dispersion (Fig. 2b). The MXene-modified fabrics are subsequently dried in a vacuum oven. This self-assembly strategy is repeated to build multilayer MXene nanosheets on the surface of the fabric and achieve MXene/3D honeycomb-structured fabrics. Owing to hydrogen bonding and electrostatic interactions (Fig. 2d), the nanosheets are uniformly anchored on the surface of the fabric fibers, as evidenced by the homogeneous distribution of Ti, C, F, and O in the elemental mapping images of the samples obtained by energy dispersive spectroscopy (EDS, Fig. 2j). In addition, the behavior of MXene-modified fabrics under intense mechanical stirring in the water also confirms their stability (Fig. S4). SEM and 3D optical microscopy show that the MXene nanosheets closely overlap with each other and form continuous folds, thereby increasing the surface roughness of the fibers and the concave structure, in turn, the light absorption efficiency of the fabric (Fig. 2i). The MXene film formed on the fiber surfaces did not affect the porosity, permeability, and water absorption of the yarns (Figs. 2c and S5, S12). XRD and Fourier infrared spectroscopy (FTIR) measurements of the pure and MXene/3D honeycomb fabrics were performed to confirm the attachment of MXene nanosheets on the fibers (Figs. 2k and S6). The (002) diffraction peak shifts from 6.46° in the pure MXene fabric to 6.04° in the MXene/3D fabric, thus, reflecting an increase in interlayer spacing from 1.351 to 1.451 nm according to the Bragg formula (2dsin*θ* = *λ*, *λ* = 0.15406 nm). The increase in interlayer spacing between MXene layers may be caused by the intercalation of water and PDDA molecules (Fig. S7) [46, 47]. Controlling the weaving parameters of the honeycomb fabric could enable the fabrication of MXene/3D honeycomb fabric samples as large as 300 × 100 mm^2^. The MXene/3D honeycomb fabric possesses excellent flexibility, bending, twisting and mechanical properties (Figs. 2e and S8), good shape tunability, and hence, excellent industrialization prospects. The industrially MXene/3D honeycomb fabric designed in this research presents a new scope for the large-scale application of solar evaporators.

**Fig. 3:**
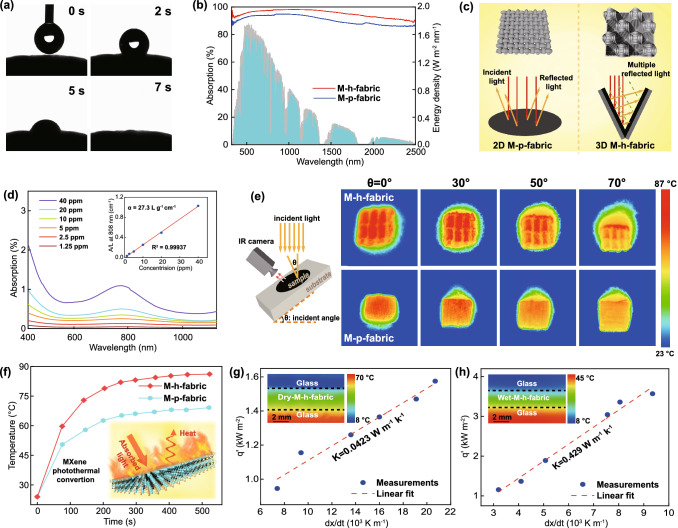
**a** Hydrophilicity of the MXene/3D honeycomb fabric. **b** Absorption spectra of the M-h-fabric and M-p-fabric (sample names: M = MXene, h = honeycomb, p = plain). **c** Schematic diagram of the reflection process of sunlight on different fabrics. The 2D plain fabric reflects sunlight only once, whereas the 3D honeycomb fabric reflects sunlight multiple times. **d** Absorption spectra of MXene solutions with different weight concentrations. Inset: Lambert–Beer law absorbance plot for absorption at 808 nm. **e** IR images of the M-h-fabric and M-p-fabric at incidence angles of 0, 30, 50, and 70°. Inset: Schematic of the incidence angles. **f** The variations of surface temperature for the M-h-fabric and M-p-fabric under the irradiation of 1 solar intensity. Inset: Schematic of the photothermal conversion of MXene. **g**–**h** Thermal conductivity of dry and wet M-h-fabrics. Insets: Representative images taken by the IR microscope

Good hydrophilicity and photothermal conversion properties are prerequisite conditions for the application of MXene/3D honeycomb fabrics as efficient and continuous solar evaporators. The water contact angles of the MXene/3D honeycomb fabric were measured to characterize its hydrophilicity. When a water droplet is dripped onto the surface of the MXene/3D honeycomb fabric, it rapidly spreads out and is absorbed by the fabric within 7 s (Fig. 3a). This finding demonstrates the good hydrophilicity of the MXene/3D honeycomb fabric, which may be attributed to the good hydrophilicity of MXene and the cotton fibers, as well as the high porosity (87%, more detail discussion as shown in Note S1) of the cotton fabric [48]. Therefore, water can quickly penetrate into the pores of the fibers for steam generation and facilitate the accumulation of salt at the edge of fabric during evaporation. The optical properties of different fabrics were measured by an ultraviolet (UV)-visible (vis)-NIR spectrophotometer (280–2500 nm). Figure S9a reveals that the reflectance of the pristine honeycomb and plain fabrics (i.e., P-h-fabric and P-p-fabric, respectively) are remarkably high (> 60%) with negligible absorbance. By comparison, the reflectance of the MXene/3D honeycomb fabric and MXene/2D plain fabrics (i.e., M-h-fabric and M-p-fabric, respectively) are much lower (< 10%) than that of pristine fabrics. Thus, MXene contributes to the excellent light absorption of the fabrics. The wet M-h-fabric is much darker in color and shows higher absorption than the dry M-h-fabric over the entire solar spectrum range investigated (Fig. S9b). This finding may be attributed to the decreased reflectance of interfacial light scattering at the liquid-to-material (i.e., wet) interface compared with that at the air-to-material (i.e., dry) interface [49]. Between the wet M-h-fabric and M-p-fabric, the former exhibits lower transmittance (~ 0%) and reflectance (~ 4%), and broader yet stronger absorbance (~ 96%) from 280 to 2500 nm; the latter presents lower light absorption (90%, Fig. 3b). Figure 3c illustrates the reason behind the high light absorption of the M-h-fabric. The planar structure of plain fabric causes light reflect directly toward the atmosphere. By contrast, the concave honeycomb structure arrays of the honeycomb fabric present nearly closed 3D prisms (8 × 10 × 8 mm^3^, length × width × height; Fig. S17). When incident light enters these prism structures, the reflected light is confined to the concave honeycomb arrays and undergoes multiple reflection/absorption processes, capturing more incident light. Moreover, the surface-roughness and textured porosity of the fabric generate multiple scattering and then multi-absorption by MXene. The UV–vis-NIR absorption spectra of MXene nanosheets (Fig. 3d) display strong absorption in the NIR region from 750 to 850 nm, similar to some noble metal nanoparticles, e.g., gold, featuring LSPR effect. According to the Lambert–Beer law (*A*/*L* = *αC*, where *A*/*L* is the standard absorption intensity at a certain *λ* and *α* is the extinction coefficient, which is an index used to determine the light-harvesting capacity of a material), the *α* of MXene at *λ* = 808 nm is 27.3 L g^−1^ cm^−1^, which is higher than those of most photoabsorption materials, for example, 13.9 for Au nanorods, 23.8 for tungsten disulfide (WS_2_) nanosheets, 3.6 for graphene oxide (GO), 24.6 for reduced GO, and 14.8 for black phosphorus [39, 50–53]. The strong absorption in the NIR region suggests the potential of the sheets as excellent photothermal conversion.

Because of the excellent light absorption properties of MXene and the honeycomb structure of the fabric, the M-h-fabric displays outstanding photothermal conversion properties. Under the vertical irradiation of 1 solar intensity, the surface temperature of the dry M-h-fabric increases rapidly to a steady-state value of 86 °C, which is much higher than that of the M-p-fabric (69 °C, Fig. 3f). This finding reflects the advantage of a concave structure of the honeycomb fabric in light trapping and thermal energy management. Considering the various positions of the sun through the day, maximum solar energy utilization at different incident angles is essential for high-efficiency solar evaporators. The surface temperatures of plain and honeycomb fabrics under the irradiation of 1 solar intensity at various incident angles are shown in Fig. 3e. Compared with that of the M-p-fabric, the surface temperature of the M-h-fabric declines by only 9 °C as the incidence angle increases from 0 to 50°. As the incidence angle increases to 70°, the surface temperature of the M-h-fabric decreases to 72 °C because of the sharp decline in the projected area of the fabric. Thus, given the excellent light absorption performance of MXene and the light-trapping structure of the honeycomb fabric, the M-h-fabric shows low dependence on the light direction and good stability for photothermal conversion.

The thermal conductivities of the dry and wet M-h-fabrics were measured and calculated (more detail discussion as shown in Note S2) to investigate the ability of confining heat to the evaporating surface for the M-h-fabric. As shown in Fig. 3g–h, the dry and wet M-h-fabrics are separately sandwiched between two slides with different temperatures, and the IR images display the temperature gradient along the thickness of the fabric layer. Because of the high porosity and concave structure (filled with air of low thermal conductivity, 0.023 W m^−1^ K^−1^) of the 3D honeycomb fabric, its thermal conductivity in the dry state is as low as 0.0423 W m^−1^ K^−1^, which is much lower than that of reported fabrics [54, 55]. For the wet M-h-fabric, the thermal conductivity is 0.429 W m^−1^ K^−1^, which is higher than that in the dry state. The high thermal conductivity of wet fabric is attributed to the filling water in the pores of fabric, but it is still lower than that of pure water (~ 0.6 W m^−1^ K^−1^). The microstructures of high porosity can help suppress local convection. Both the reduced thermal conductivity and the suppressed convection contribute to the reduced heat loss. Overall, the low thermal conductivity of M-h-fabrics results in confinement of heat to the evaporator surface. The good wettability, effective light absorption, porous structure with vapor channels, excellent mechanical properties, and low thermal conductivity of the M-h-fabrics render them ideal evaporators for solar vapor generation.

**Fig. 4:**
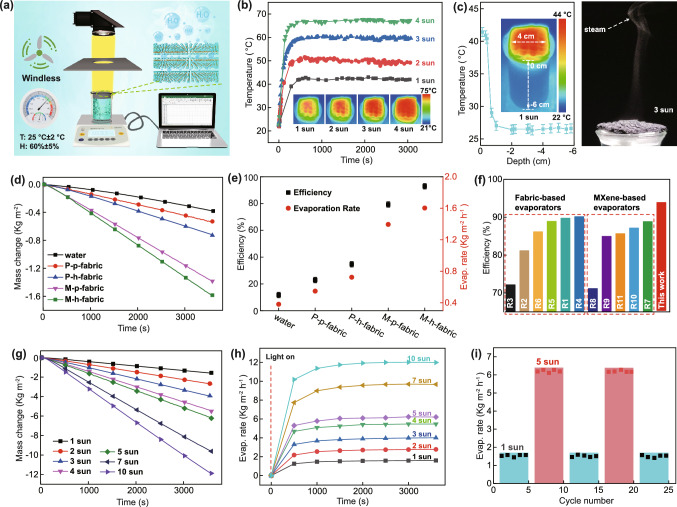
**a** Schematic diagram of the system used to test the solar vapor generation performance of the evaporator. **b** Variations in the surface temperature of the honeycomb fabric with irradiation time under different light intensities. Inset: IR image of the honeycomb fabric surface. **c** Longitudinal temperature distribution of the evaporator under 1 solar intensity (left panel) and water vapor generated under 3 solar intensities (right panel). **d** Changes in the evaporation mass of water, P-p-fabric, P-h-fabric, M-p-fabric, and M-h-fabric under the illumination of 1 solar intensity (sample names: P = pristine, M = MXene, h = honeycomb, p = plain). **e** Evaporation rate and efficiency of water, P-p-fabric, P-h-fabric, M-p-fabric, and M-h-fabric under the illumination of 1 solar intensity. **f** Comparison of the evaporation efficiency of the M-h-fabric prepared in this work with those of previously reported evaporators. **g** Variations in the evaporation mass of the M-h-fabric over time under different solar intensities. **h** Evaporation rate of the M-h-fabric under different solar intensities. **i** The cycle stability of evaporation performance for the M-h-fabric under different solar intensities. Each cycle was sustained for 1 h

Given the excellent photothermal effects of the MXene/3D honeycomb fabric, the solar evaporation performance of the evaporator system (M-h-fabric + polystyrene foam + cotton fiber rods) was investigated using a homemade real-time measurement system in the laboratory (Fig. S10). A schematic diagram of the water evaporation measurement system is shown in Fig. 4a. The change in water mass generated by the vapor was measured in real time using an electronic analytical balance, and the surface temperature of the solar vapor generation device was recorded by an IR camera. Because a photothermal vapor generation unit with excellent thermal management performance is essential for high conversion efficiency, the thermal localization ability of the evaporator under solar irradiation was studied in detail. The surface temperatures of the solar vapor generation device under the irradiation of 1–4 solar intensities are shown in Fig. 4b. As the solar intensity gradually increases from 1 to 4, the surface temperature of the device increases rapidly and then stabilizes within 5 min; specifically, the surface temperature increases from 41 to 67.7 °C. By comparison, the temperature of pure water changes little. As shown in Fig. 4c, the surface temperature of the M-h-fabric rapidly increases from 23 to 41 °C under the irradiation of 1 solar intensity. However, the temperature of the bulk water barely changes, showing an increase in only approximately 0.3 °C within 60 min under the irradiation of 1 solar intensity. Indeed, even under the irradiation of five solar intensities, bulk water shows a temperature change of only approximately 2.6 °C. Thus, our evaporator consisting of low thermal conductivity honeycomb fabric at the top and polystyrene foam at the bottom presents outstanding thermal localization performance.

The highly localized heating performance of our evaporator favors efficient vapor production. For example, steam is clearly observed under the irradiation of three solar intensities (Fig. 4c), and the amount of steam increases with increasing irradiation (Fig. S11 and Video S1), thereby indicating the advantages of interfacial heating in our evaporator. The outstanding thermal management of our evaporator may be attributed to the combination of the honeycomb fabric with low thermal conductivity and polystyrene foam. Vertical water transport channels are also restricted in the cotton fiber rods, which further minimizing heat transfer loss. In particular, honeycomb fabrics with periodic arrays of concave patterns generally exhibit higher temperatures at the bottom of the concave valley than that at apex fold within a honeycomb cell. The fact is attributed to the multiple reflection and absorption of the incident light in the bottom of the concave valley. In addition, the low temperature at the apex is due to the high level of vigorous evaporation. Due to the temperature difference between the bottom and apex of the concave valley, the water evaporation rate of the honeycomb structure evaporator is further facilitated by thermocapillary force or temperature gradient-induced Marangoni effect [56]. This feature causes the heat within honeycomb fabric to flow from the bottom to the apex of the concave valley. The heat dissipated from the bottom of the hive by radiation is spontaneously absorbed and reused by the relatively cooler top of the same hive cell as it moves upward. In addition, when water vapor diffuses from the hotter bottom to the colder top, some heat from the water molecules can be transferred to the top by conduction, convection, or radiation and then used for further evaporation. This mechanism enables the reuse of waste heat and more efficient of heat utilization (Note S3).

The weight loss of water under solar irradiation (incident angle 0°) was measured to evaluate the steam generation performance of our evaporator. As shown in Fig. 4d, as the irradiation time under 1 solar intensity increases, the mass of the evaporated water linearly increases. The evaporation rate of the M-h-fabric is 1.62 kg m^−2^ h^−1^, which is approximately 1.2 times of the evaporation rate of the M-p-fabric (1.39 kg m^−2^ h^−1^) and 4.3 times that of pure water (0.38 kg m^−2^ h^−1^). Similarly, the evaporation rate of the P-h-fabric without MXene is 0.73 kg m^−2^ h^−1^; this value is also higher than that of the P-p fabric (0.55 kg m^−2^ h^−1^). The solar evaporation efficiency (*η*) of our evaporator was calculated (more detail discussion as shown in Note S4) to evaluate the capacity of energy utilization. As shown in Fig. 4e, the energy conversion efficiencies obtained are in agreement with the trend of water evaporation rate. The solar evaporation efficiency of the M-h-fabric may be as high as 93.5%, which is much larger than that of the M-p-fabric (79.5%). The value is much higher than that of all previously reported MXene-based and fabric-based evaporators and the evaporation rate and evaporation efficiency are among the highest of all solar absorbers (Fig. 4f and Table S1) [11, 34, 36, 38, 55, 57–62]. This result demonstrates the advantage of a honeycomb structure in energy utilization. The excellent evaporation rate and energy conversion efficiency of the M-h-fabric could be mainly attributed to its large specific surface area (i.e., real water evaporation area), which is approximately 2.06 times greater than that of M-p-fabric (more detail discussion as shown in Note S5). Such a large specific surface area endows the M-h-fabric with a large surface area for sunlight absorption and water evaporation. The concave structured in the honeycomb fabric improved the light-harvesting and thermal management, thus improving its solar-thermal conversion efficiency. Simultaneously, the strong pumping ability of the cotton rods can form sufficient and appropriate water film on the surface of fabrics for constant evaporation, i.e., the water replenishment rate matches the water evaporation rate (Figs. S12 and S13), resulting in the constant water evaporation rate (Fig. S14a). However, if the speed of water supplementation cannot satisfy the speed of water evaporation, the dehydrated surface of the fabric will result in the decrease of the water evaporation rate (Fig. S14b) and the increase in the surface temperature (Fig. S15). Therefore, our evaporator system has balanced water pumping rate and interfacial evaporation rate for high water evaporation efficiency [56, 63–65]. Further, the water evaporation rate of the evaporator decreases with the increasing angle of solar illumination (Fig. S16), i.e., the reduced solar flux resulted in the lower water evaporation rate, further proving the rational solar illumination angle in our evaporator [66]. The water evaporation rate of our M-h-fabric-based evaporator could be further improved by increasing the irradiation power density to achieve evaporation rates of 2.78, 4.01, 5.47, 6.21, 9.67, and 11.99 kg m^−2^ h^−1^ under the irradiation of 2, 3, 4, 5, 7, and 10 solar intensities, respectively (Fig. 4g, h). These findings demonstrate that our evaporator still performed well under high light irradiation intensities. More importantly, the evaporator reveals a stable evaporation rate even after 25 cycles, each lasting 60 min, under different light illumination intensities (Fig. 4i). Thus, our evaporator shows an excellent cycling stability for repeated use. In summary, the M-h-fabric developed in this work presents feasibility and outstanding water evaporation performance.

**Fig. 5:**
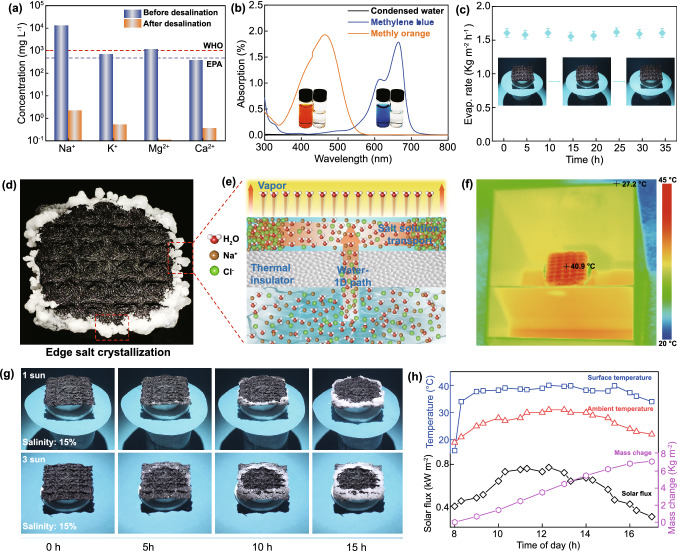
**a** Concentrations of major cations in real seawater (Yellow Sea, China) before and after desalination. **b** UV–vis absorption spectra of methylene blue (MB) and methyl orange (MO) solutions before evaporation and the corresponding condensed water after evaporation. **c** Comparison diagram of a honeycomb fabric-based evaporator before and after 35 h of continuous evaporation. **d** Digital image of salt accumulation at the edge of the evaporator. **e** Schematic diagram of water evaporation, water transport, and salt accumulation in a solar steam generator. **f** IR image of the solar after 20 min of solar irradiation. **g** Digital image of salt accumulation under the irradiation of 1 and 3 solar intensities with salt concentration of 15%. **h** Variations in solar intensity, honeycomb fabric surface temperature, ambient temperature, and evaporation mass with time from 8:00 to 17:00 (Nov. 12, 2020; Qingdao, China)

The vapor generated by light irradiation was collected by a homemade device in our laboratory to evaluate the desalination performance of our evaporator (Fig. S20). The concentrations of four primary ions (Na^+^, K^+^, Ca^2+^, and Mg^2+^) found in water samples of the Yellow Sea before and after desalination were measured by inductively coupled plasma-optical emission spectrometry (ICP-OES). As shown in Fig. 5a, the ion concentration of the seawater significantly decreases by 3–4 orders of magnitude after desalination, whose level is much lower than the drinking water quality standards of the World Health Organization and the US Environmental Protection Agency. We prepared NaCl solutions of different concentrations of 3.5, 10, 15, and 21 wt% for water desalination and found that the Na^+^ concentrations of the steam water collected from these solutions are approximately 1.6, 1.7, 1.8, and 2.1 mg L^−1^ (Fig. S21), respectively. These findings demonstrate the excellent performance of our evaporator for water desalination even with highly concentrated brine. Water evaporation was attempted using wastewater bearing the dyes methyl orange and methylene blue as simulated pollutants. The strong absorption peaks of methyl orange (~ 465 nm) and methylene blue (~ 665 nm) are nearly completely eliminated in the condensed water (Fig. 5b). The above results indicate that the M-h-fabric-based evaporator has excellent performance in the water purification of seawater and sewage water.

The salt resistance of the M-h-fabric-based evaporator for continuous steam generation was investigated. The water evaporation rate of the evaporator changes slightly under the irradiation of 1 solar intensity for 35 h (3.5% NaCl solution, Fig. 5c). After desalination, the evaporator surface remains clean, and only a very small amount of salt crystalloids is noted at the edge of the fabric. These results indicate the good salt resistance of our evaporation system. Interestingly, under the illumination of 1 solar intensity for 15 h, large amounts of salt crystals accumulate only at the edge of the fabric when the salt concentration is increased to 21%; very obvious salt rings are observed in the fabric in this case (Fig. 5d). Moreover, the harvested salt will not dissolve back in the simulated day-night situation (light up for 8 h and then light off for 12 h, Fig. S22). The accumulated salt particles fall off from the evaporator with a light tap (Fig. S23), indicating that our evaporator is capable of continuous water evaporation and simultaneous salt harvesting. The mechanism of salt crystallization is shown in Fig. 5e. The superior hydrophilicity and capillary forces of the cotton fiber rods allow them to transport salty water from the bulk solution to the center of the M-h-fabric. Given the good hydrophilicity of the fabric, the salt solution is transported from its center to edge. During water transport, steam is generated from the surface of the fabric because of the photothermal conversion effect of MXene. As a result, as the salt solution approaches the edge, the salt concentration gradually increases, and salts crystallize at the edges of the fabric. The presence of salt concentration gradients on the evaporator surface also induces a Marangoni effect. The surface tension gradient along this interface generates tangential shear, creating a slip velocity that drives the flow of seawater from the region with low surface tension to the region with high surface tension for the interface, i.e., from the center to the edge of the evaporator, ensuring a seawater flow rate that facilitates evaporation. Therefore, in the case of honeycomb fabric-based evaporator system, the concave structure of the honeycomb fabric surface introduces not only a temperature gradient in response to solar irradiation, but also a solutal gradient, which activates Marangoni convection and accelerates the flow of water, contributing to its evaporation and salt harvesting [67, 68]. The location of salt crystallization is closely related to the water evaporation ability. As shown in Fig. 5g, under a salinity of 15%, the salt crystallizes at the edge of the fabric under the irradiation of 1 solar intensity (Video S2), while salt crystalizes at a short distance from the center of the fabric under the irradiation of 3 solar intensities. The difference in these crystallization locations may be attributed to the fact that the water evaporation ability of the fabric is much higher under the irradiation of 3 solar intensities. Once the concentration of salt exceeds its dissolution limit at a point (the distance from this point to the center is less than the radius of the evaporator), the salt gradually accumulates from the point to the center of the evaporator. In addition, the water transport path also influences the position of salt crystallization on the evaporator. For example, the insulation foam is wrapped by the honeycomb fabric, and the edge of the fabric is immersed in the water, thus forming a 2D plane water transfer path for the evaporator (Fig. S24). Because solution transfer occurs from the edge to the center of the evaporator, the concentration of NaCl in the remaining solution gradually increases when it approaches to the center during prolonged evaporation, and the salt accumulates on the surface of the fabric, thereby reducing the evaporation rate of the device.

The outdoor water evaporation experiments were conducted using a homemade evaporator under natural sunlight to explore the practical applications of our device. A model house with a slanted roof was fabricated from a transparent acrylic plate to achieve the evaporation and collection of water (Fig. S25). An enlarged evaporator (honeycomb fabric with length × width, 8 × 6 cm^2^) was prepared, and the evaporation experiment was performed on a sunny day from 8:00 to 17:00 (November 12, 2020; maximum air temperature, 30 °C; Qingdao, China). The time-dependent solar flux, evaporator surface temperature, ambient temperature, and water mass changes were monitored, and the results are displayed in Fig. 5h. The solar flux increases from 0.4 kW m^−2^ at 8:00 to 0.7 kW m^−2^ at 10:00, stabilizes at ~ 0.7 kW m^−2^ from 10:00 to 14:00, and then decreases to 0.4 kW m^−2^ at 16:00. The surface temperature of the wetted evaporator increases with increasing solar flux, and the maximum temperature measures is 40.9 °C, which is approximately 10 °C higher than the ambient temperature (Fig. 5f, h). The water evaporation rate of the evaporator is clearly proportional to the solar flux, and the maximum water evaporation rate increases to 0.99 kg m^−2^ h^−1^ at the peak solar irradiation intensity. From 8:00 to 17:00, the steam generated by our evaporator is as high as 6.9 kg m^−2^, which is sufficient for three persons’ daily drinking water consumption. At the same time, there is no salt accumulation on the evaporator surface. Moreover, the resistance of the collected purified water increases from 81.24 KΩ to 1.272 MΩ after purification, thereby demonstrating the excellent seawater purification capability of our honeycomb fabric evaporator (Fig. S26). These results of these outdoor experiments demonstrate the great potential of our M-h-fabric-based evaporator for steam generation and outstanding solar desalination in long-term practical applications.

## Conclusion

In conclusion, inspired by nature, we have developed a facile and scalable method to fabricate solar steam evaporator based on MXene-decorated 3D honeycomb-structured fabric, which successfully achieved a high solar efficiency of up to 93.5% and an excellent evaporation rate of 1.62 kg m^−2^ h^−1^ under one sun illumination. Additionally, the evaporation rate of the MXene/3D honeycomb fabric evaporator remained stable during 25 cycles and 35 consecutive hours of seawater evaporation, where a confined salt solution transferring path enabled the salt crystals spatially isolating from the surface of evaporator and thus ensured its long-term usage stability and salt harvesting. The high solar efficiency is attributed to the integrated optimization and synergistic roles of multifunctional layers, including MXene-coated 3D honeycomb fabric surface with periodic concave as effective light absorbers, a low thermal conductivity honeycomb fabric supported by the insulating foam as thermal barrier, 1D hydrophilic fibrous rods with strong capillary force located perpendicularly to the center of highly hydrophilic honeycomb fabric as water transport path and salt rejection, and the porous of fabric as vapor channels, which results in simultaneous achievements of superior light absorption, thermal management, water transport and salt harvesting performance. The low-cost, portable, flexibility, scalable MXene/3D honeycomb fabric solar evaporator offers new opportunities for desalination, wastewater purification, and recovery of valuable salts from water.

## Supplementary Information

Below is the link to the electronic supplementary material.

Supplementary file1 (MP4 7355 kb)

Supplementary file2 (MP4 8604 kb)

Supplementary file3 (PDF 4208 kb)
