# Supplementary material for: Nature Inspired MXene-Decorated 3D Honeycomb-Fabric Architectures Toward Efficient Water Desalination and Salt Harvesting
Source: Nanomicro Lett. 2021 Dec 4;14:10. doi: 10.1007/s40820-021-00748-7 (PMC8643288; doi:10.1007/s40820-021-00748-7)
Supplement: Supplementary file 3 — Supplementary file3 (PDF 4208 kb) [file 40820_2021_748_MOESM3_ESM.pdf]

Supporting Information for

Nature Inspired MXene-Decorated 3D Honeycomb-Fabric Architectures toward Efficient Water Desalination and Salt Harvesting

Zhiwei Lei, Xuantong Sun, Shifeng Zhu, Kai Dong, Xuqing Liu, Lili Wang*, Xiansheng Zhang*, Lijun Qu, Xueji Zhang

S1 Experimental

Note S1 Measurement of the effective porosity of the 3D honeycomb fabric

Under the dry state, the fabric was weighed with an analytical balance. After soaking in water for one hour to reach a fully wetted state, it was weighed again. The effective porosity of the fabric can be calculated according to the Eq. (S1).

$$\mathbf{Q}(\%) = \frac{(\mathbf{m_2}-\mathbf{m_1})/\rho_2}{\mathbf{m_1}/\rho_1 + (\mathbf{m_2}-\mathbf{m_1})/\rho_2} \qquad \textbf{(S1)}$$

Where m_1 and m_2 are the weight of the fabric before and after wetting (mg), respectively, and ρ_1 and ρ_2 are the densities of cellulose (1.5 g cm^{-2}) and water (1 g cm^{-2}), respectively. In order to minimize the experimental error, the fabric was measured three times and the average data was used.

Note S2 Thermal conductivity measurements of dry/wet 3D honeycomb fabric

The thermal conductivity of the dry/wet MXene/3D honeycomb fabric was measured by sandwiching the material between two 1 mm thick glass slides [S1]. The "sandwich" was placed between a heat source (JF-956, China) and a cold source (ice water bath). The temperature distribution along the cross section of the sandwich structure was monitored using an IR camera. The thermal conductivity is calculated based on Fourier's law (Eq. 2).

$$\mathbf{q}' = \mathbf{K}\frac{\Delta \mathbf{T}}{\Delta \mathbf{X}} \qquad \textbf{(S2)}$$

Since the thermal conductivity (K) of the glass slide (1.05 W m^{-1} K^{-1}) is known, ΔT is the temperature difference, and ΔX is the distance difference, the heat flux q' per unit area was calculated accordingly. Assuming that the sample and the slide have the same heat flux, the thermal conductivity of the fabric was calculated.

Note S3 Calculation of the theoretical overall heat loss and efficiency

Theoretical overall heat loss and efficiency were calculated to explore the functionality

of the MXene modified honeycomb fabric evaporator. Using the Stefan-Boltzmann equation, Newton's law of cooling and heat flux for a large amount of water, a detailed heat loss analysis was performed as shown in Eqs. (3) and (4) [S4-S7]:

$$\mathbf{P_{environment} = \varepsilon\sigma(T_2^4 - T_1^4) + h(T_2 - T_1) + \frac{cm\Delta T}{At}} \quad \text{(S3)}$$

$$\boldsymbol{\eta} = \frac{\mathbf{1000 - P_{environment}}}{\mathbf{1000}} \quad \text{(S4)}$$

Here, ε is the emissivity (i.e., 0.98), σ is the Stefan-Boltzmann constant (i.e.,5.67×10^{-8} W m^{-2} K^{-4}), h is the convective heat transfer coefficient of about 5 W m^{-2} K^{-1}, C is the specific heat capacity of water (i.e., 4.18 J C^{-1} g^{-1}), and m (i.e.,30 g) is the weight of water. T_2 (41 °C) is the mean surface temperature evaporator steady state temperature for about 1 hour and T_1 is the environment temperature surrounding the sample. According to previous reports [S5, S6], the heat gathered on the solar absorber is exchanged with the surrounding solar steam in a small area rather than dissipated into the environment. In particular, since that the top surface of the absorber is surrounded by water and hot steam, the temperature of the environment adjacent to the top of the absorber is close to that of the absorber and steam (Fig. S19). In this work, a thermocouple was used to measure the vapor temperature top of the sample, and T_1 was measured to be about 39.0 °C (about 7 mm above the center of the device). ΔT/t is the change in total water temperature during the test (i.e., 0.3 °C within t seconds (3600 s)), and A is the area of water transfer. According to the equation (S3 and S4), the power of heat loss is recalculated to be approximately 32.8 W m^{-2}. Most absorbed solar energy is still used to evaporate the water sheet on top of the absorber surface rather than being lost through these channels. The theoretical efficiency of solar energy utilization is 96.72%.

Note S4 Calculation of solar evaporation efficiency

The solar evaporation efficiency of samples under the solar simulator, which is defined as the Eq. (5) [S2]:

$$\boldsymbol{\eta} = \mathbf{mh_{LV}/C_{opt}q_i} \quad \text{(S5)}$$

Here, m is the evaporation rate, h_{LV} is the total enthalpy including sensible heat and the liquid-vapor phase change, C_{opt} denotes the optical concentration, and q_i is the solar radiation. It should be noted that m should be the difference between the total evaporation rate and the natural volatilization rate without light irradiation. The natural volatilization rate of MXene/3D honeycomb fabric and MXene/2D plain fabric were measured to be 0.22 and 0.20 kg m^{-2} h^{-1}, respectively. Therefore, the light induced evaporation rates of MXene decorated honeycomb fabric under 1 sunlight was 1.62 minus 0.22 kg m^{-2} h^{-1}, while the light induced evaporation rates of MXene modified plain fabric was 1.39 minus 0.20 kg m^{-2} h^{-1}.

The variation of h_{LV} at different temperatures and its relationship can be described by

an approximate Eq. (S6) [S3]:

$$\mathbf{h_{LV} = \alpha + \beta T + \gamma T^{1.5} + \delta T^{2.5} + \varepsilon T^{3}} \quad \textbf{(S6)}$$

where α = 2500.304, β = −2.2521025, γ = −0.021465847, δ = 3.1750136×10^{-4}, ε = −2.8607959 ×10^{-5} are constants and T is the temperature (°C). Thus, the h_{LV} of MXene/2D plain fabric with the surface temperature of 45 °C is 2394.19 kJ kg^{-1}, and the h_{LV} of MXene/3D honeycomb fabric with the surface temperature of 41 °C is 2403.78 kJ kg^{-1}. In the wet state, evaporation on the large surface area of the honeycomb fabric is more intense than on the liquid/vapour interface of a plain fabric, requiring more evaporative water energy and thus lowering the surface temperature. Thus, the evaporation efficiency of MXene/3D honeycomb fabric in one sunlight irradaition is 93.5% while MXene/2D plain fabric is 79.5%.

Note S5 Calculation method of 3D honeycomb fabric evaporator performance

It is worth mentioned that the area for the calculation of water evaporation rate is the light-receiving area, rather than the actual evaporation area. We assume that the concave structure of the single honeycomb is similar to a quadrangular pyramid (Fig. S18). The bottom area of the quadrangular pyramid corresponds to that exposed to sunlight for the fabric. The bottom rectangle is about 10 mm in length and 8 mm in width, and the light-receiving area is calculated by Eq. (S7):

$$\mathbf{S_{receiving\ light} = ab = 10 \times 8 = 80mm^2} \quad \textbf{(S7)}$$

The height of the quadrangular pyramid is 8 mm. The area of the quadrangular pyramid is calculated by Eq. (S8):

$$\mathbf{S_{actual\ evaporation\ area} = a\sqrt{\frac{b^2}{4} + h^2} + b\sqrt{\frac{a^2}{4} + h^2} = 10 \times \sqrt{\frac{8^2}{4} + 8^2} + 8 \times \sqrt{\frac{10^2}{4} + 8^2} = 165mm^2} \quad \textbf{(S8)}$$

Hence, we can calculate that the area of actual evaporation is about 2.06 times that of receiving light.

S2 Supplementary Figures

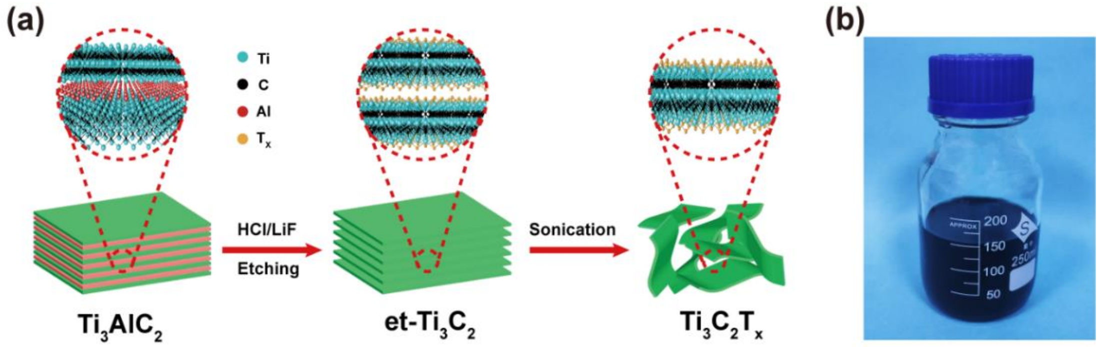

Fig. S1 **a** Schematic illustration of preparation process of MXene. **b** Aqueous solution of MXene nanosheets

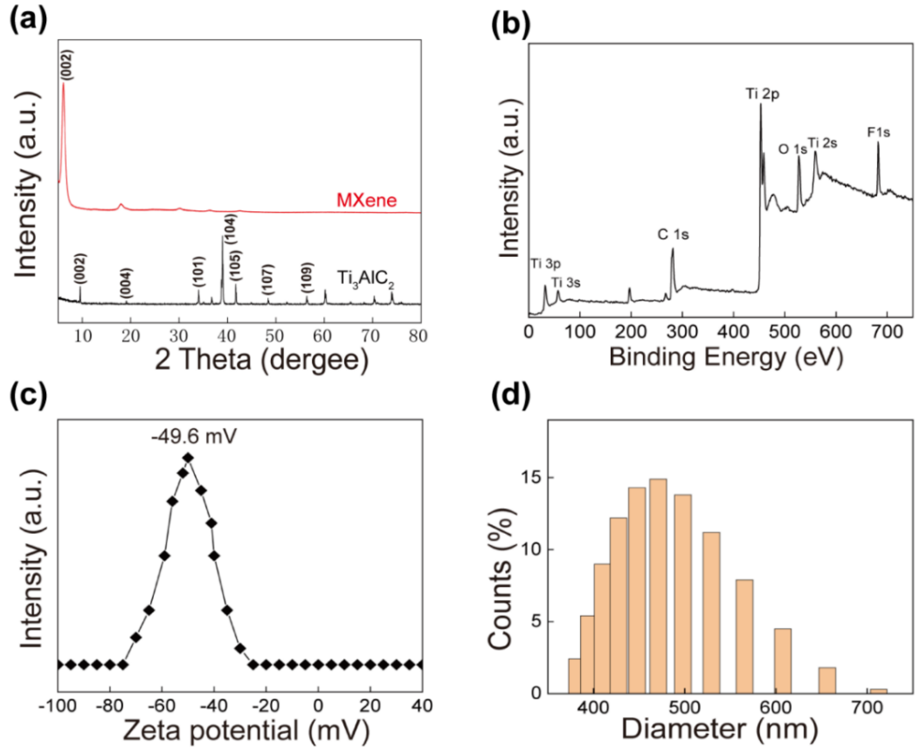

Fig. S2 **a** XRD patterns of Ti_3AlC_2 and MXene. **b** XPS spectra of MXene. **c** Zeta potential at pH of 6.2. **d** Size distribution of MXene nanosheets

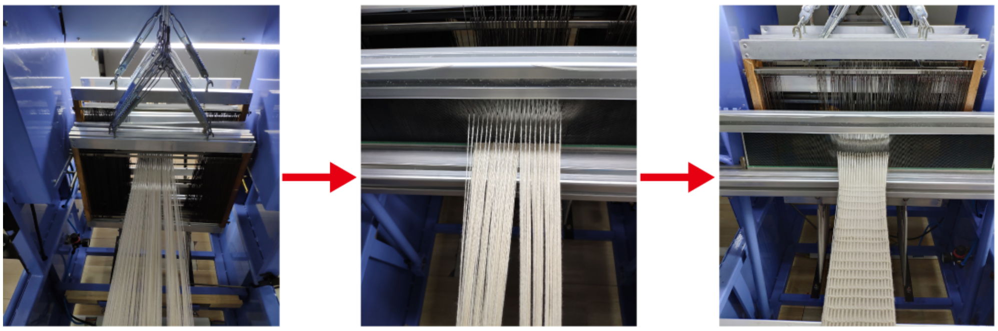

Fig. S3 The preparation procedure of 3D Honeycomb fabric by weaving machine in laboratory

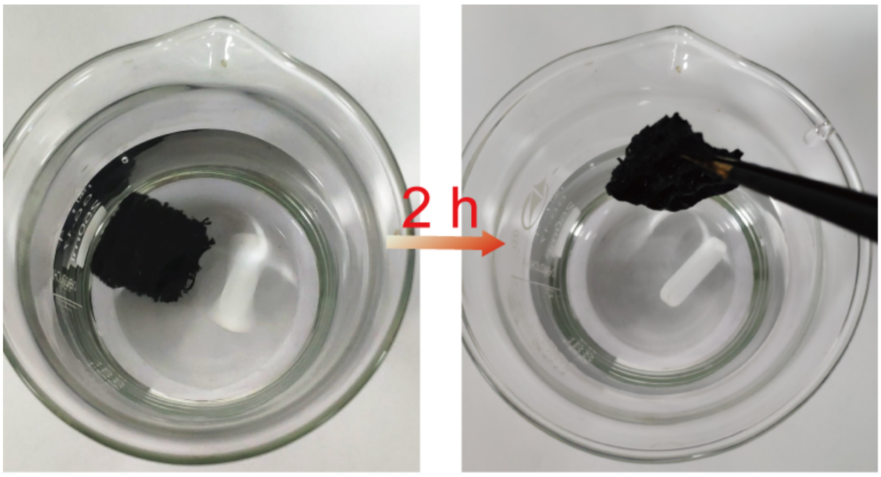

Fig. S4 The display of the MXene-modified fabrics under stirring water for 2 h

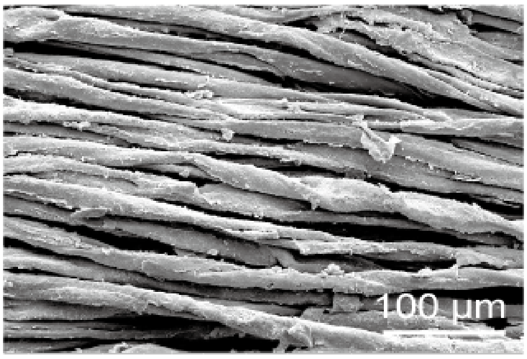

Fig. S5 SEM images of the cotton yarn surface coated with MXene flakes

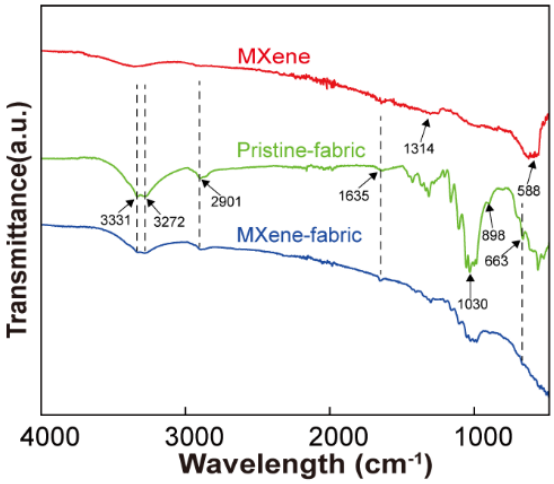

Fig. S6 FTIR showed that MXene had two characteristic peaks at 1314 and 588 cm^{-1}, corresponding to the C-F and -OH groups on the MXene surface, respectively. The characteristic absorption bands of cellulose at 2901 (C-H stretching), 1635 (-OH bending) and 663 cm^{-1} (-OH out-of-bending) were observed in the FTIR spectra of MXene/3D honeycomb fabric after impregnation treatment, further indicating that MXene was successfully compounded onto the fiber [S8].

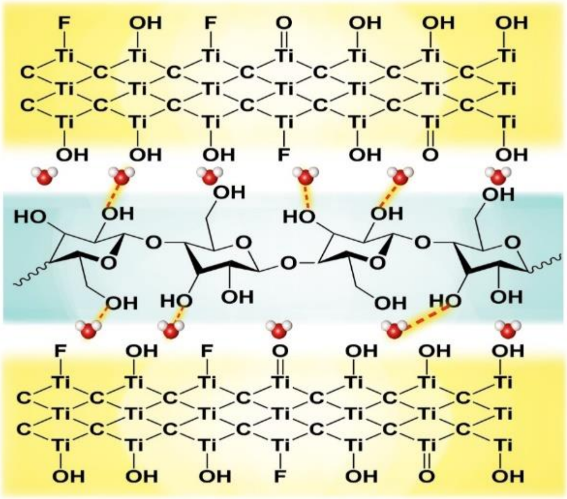

Fig. S7 Schematic illustrations of the enlarge d-spacing of 2D MXene nanosheets induced by the adsorbed water molecules

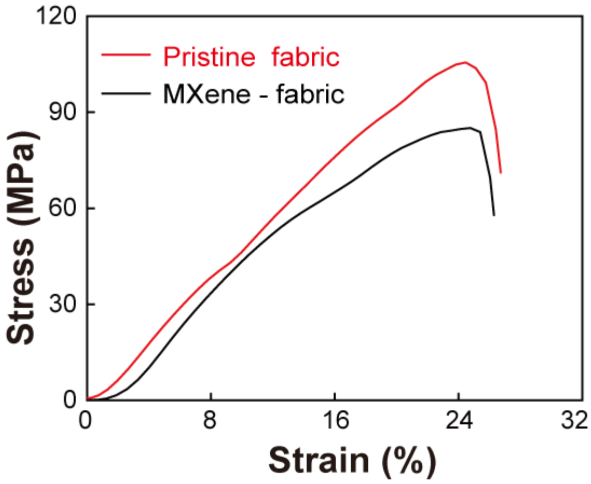

Fig. S8 Stress-strain curves of pristine fabric and MXene-fabric

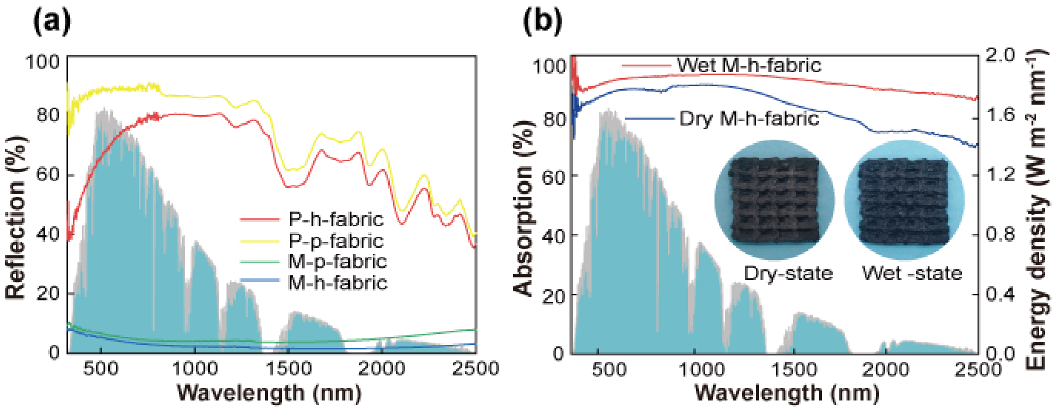

Fig. S9 a Diffuse reflection spectra of P-h-fabric, P-p-fabric, M-p-fabric, M-h-fabric in the wavelength range of 280-2500 nm. **b** Absorption spectra of dry and wet M-h-fabric samples. The inset shows that the optical images of the dry and wet states

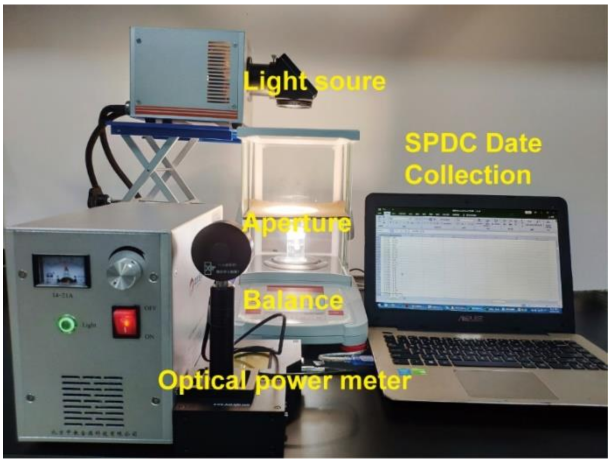

Fig. S10 The image of the water evaporation device. The size of the spot is controlled through an aperture to ensure that it equals to the size of the absorber. The evaporative mass is measured by a balance with high precision. The data is then transferred to a computer to evaluate the evaporation rate and the solar thermal conversion efficiency

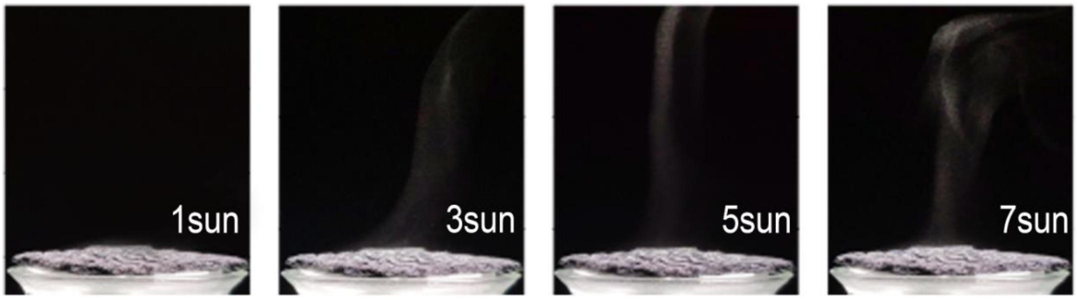

Fig. S11 Digital images of steam coming from the 3D Honeycomb fabric surface under various light intensity of 1, 3, 5, and 7 solar intensities

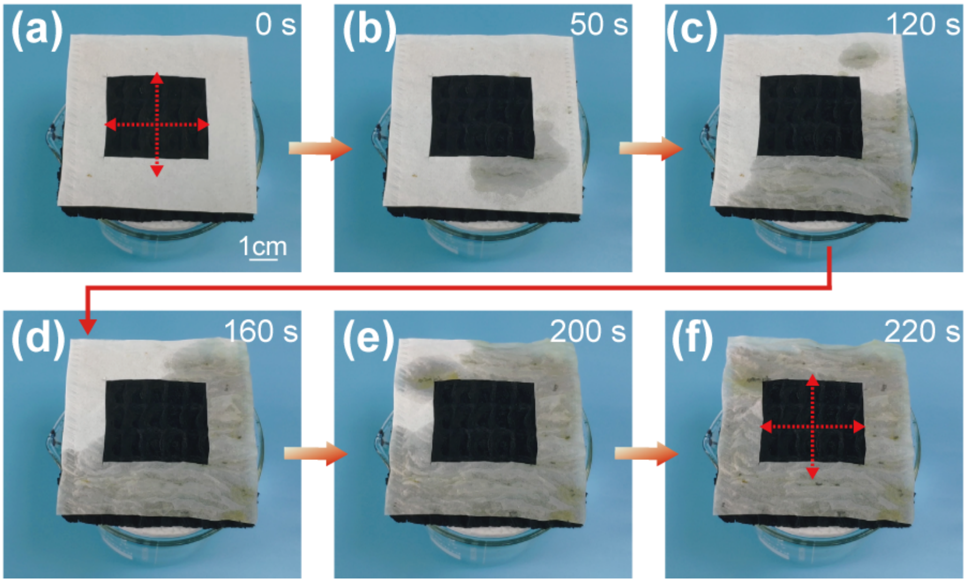

Fig. S12 The wetting process of the paper on the surface of the MXene-modified fabrics

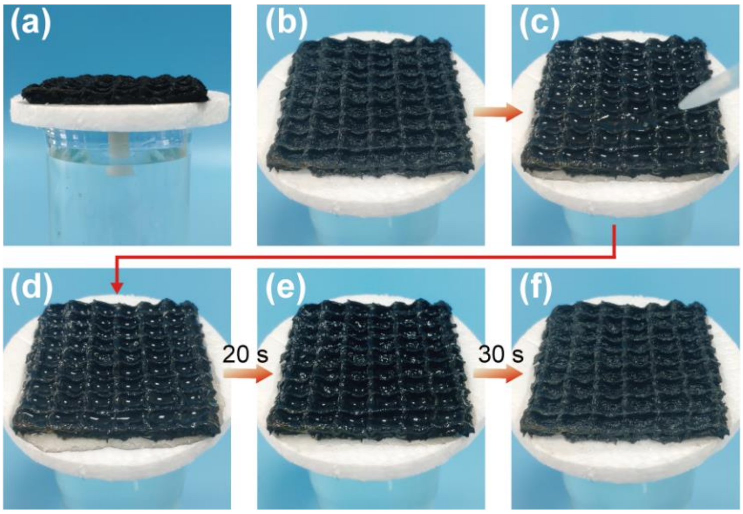

Fig. S13 The variation of the water film on the surface of the fabric with dripping water on top of the it

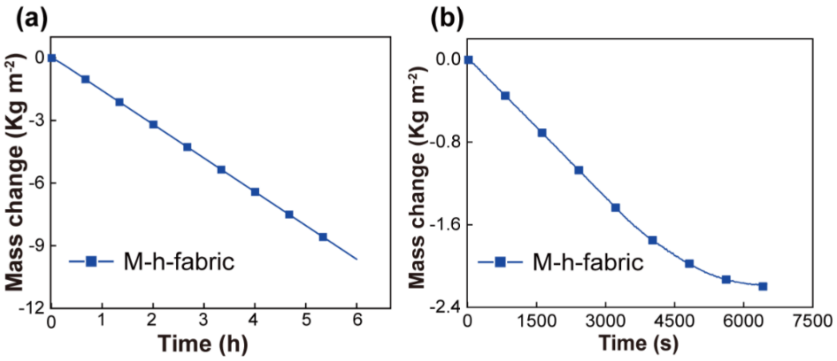

Fig. S14 a Time-dependent weight loss of water under the illumination of 1 solar intensity with effective water supply. b Time-dependent weight loss of water under the illumination of 1 solar intensity with the absence of adequate water

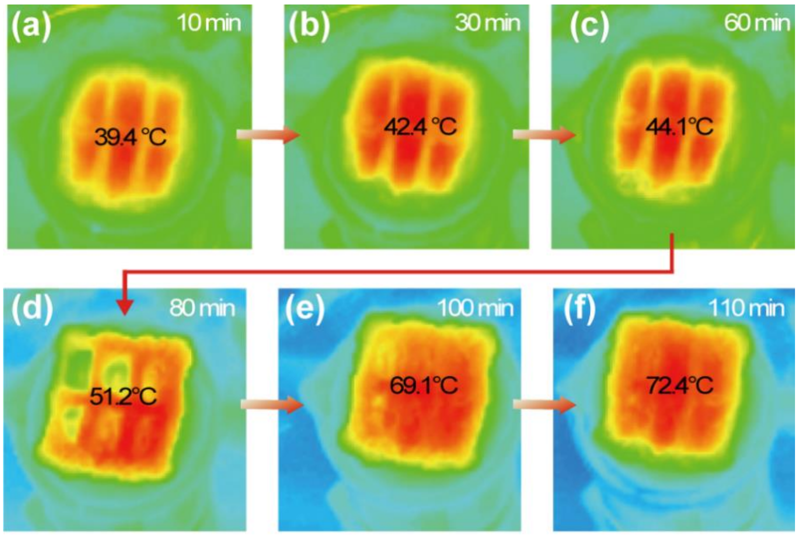

Fig. S15 The surface temperature variation of the fabric during evaporation under insufficient water supply

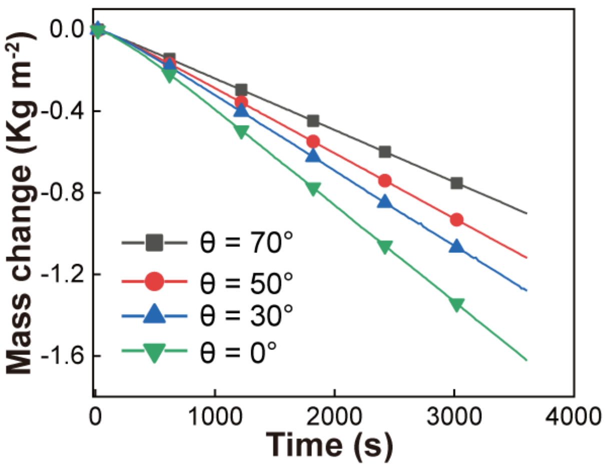

Fig. S16 The water mass loss of the evaporator at different angle under 1 solar intensity

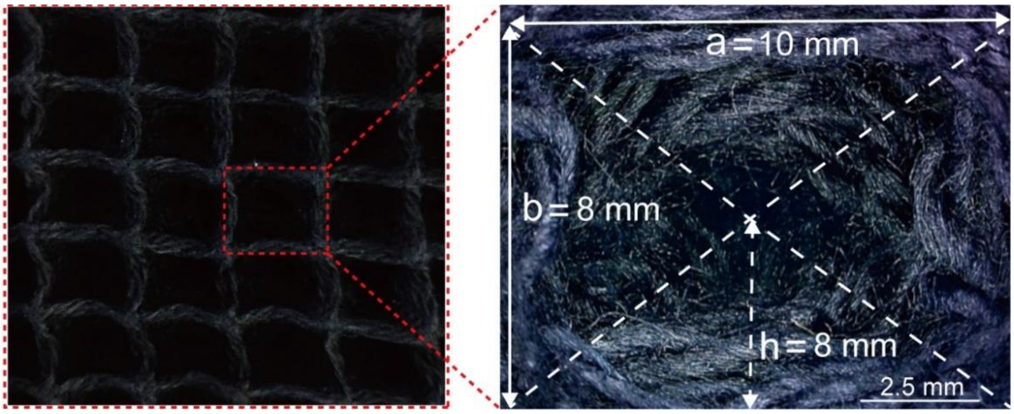

Fig. S17 The digital image of honeycomb fabric and the individual concave captured by ultra-deep field microscope

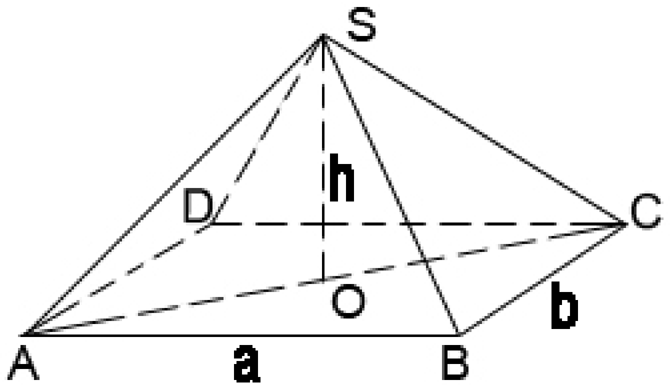

Fig. S18 Schematic diagram of a 3D honeycomb unit

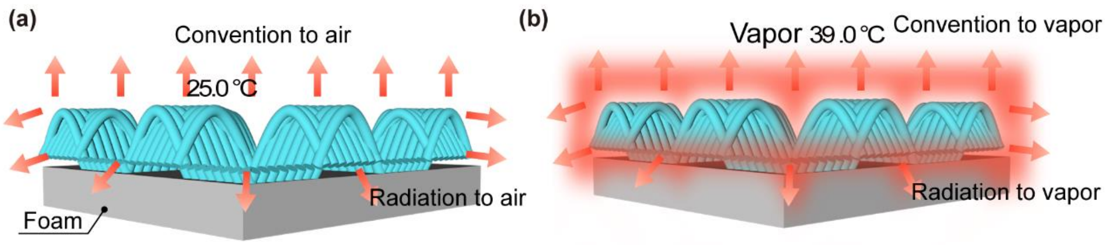

Fig. S19 a The heat loss to environment and. b to the solar vapor calculated by Stefan-Boltzmann formula and Newton's law

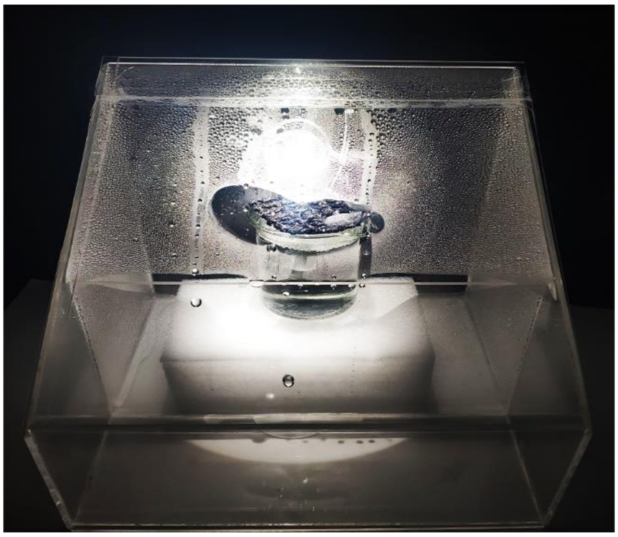

Fig. S20 Digital images of vapor condensation device

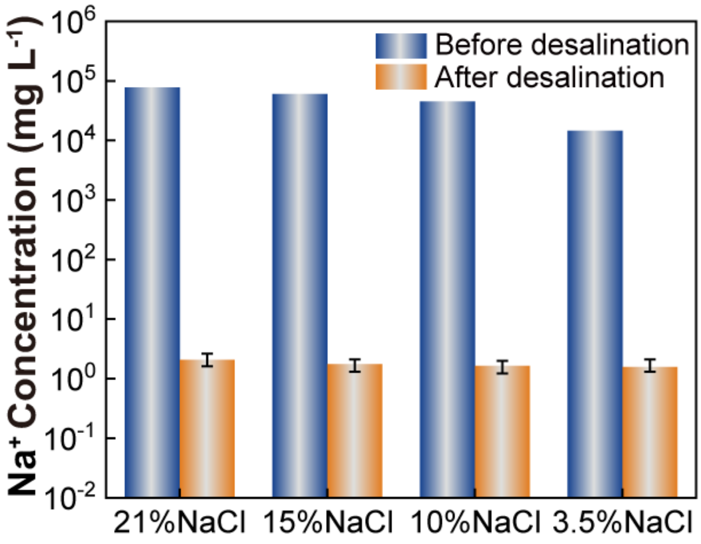

Fig. S21 Na^{+} concentrations of the original NaCl solutions and the corresponding fresh water after desalination

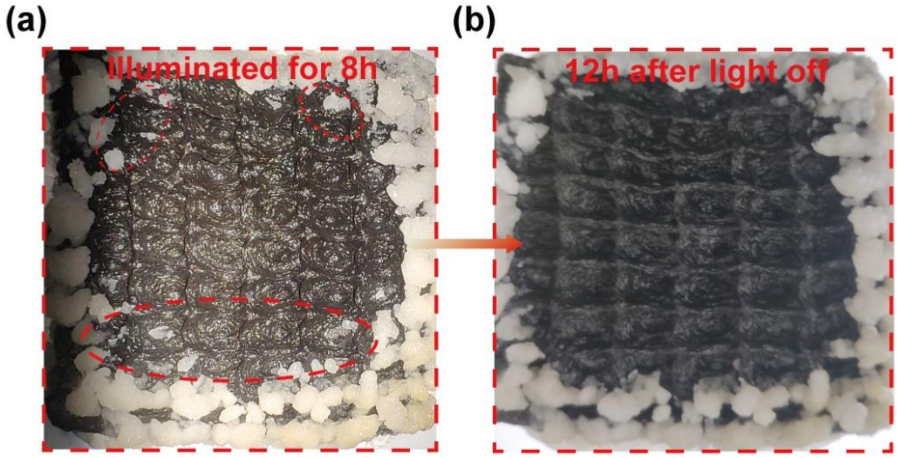

Fig. S22 a The digital image of the salt accumulation after the continuous exposure for 8h under 1 solar intensity. b The digital image of the fabric without solar illumination for 12 h

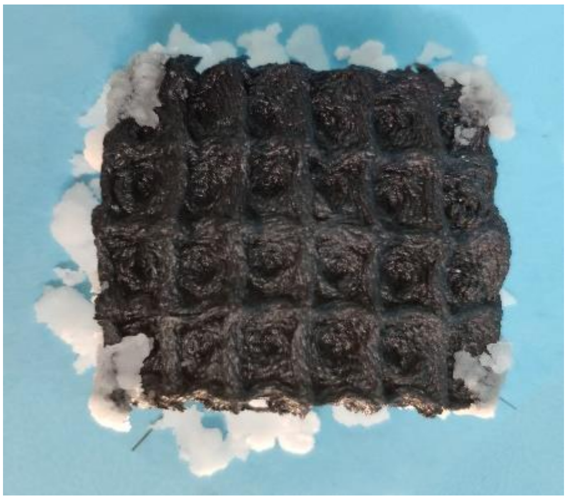

Fig. S23 The salt particles can easily fall off the edge from the evaporator under the action of external force

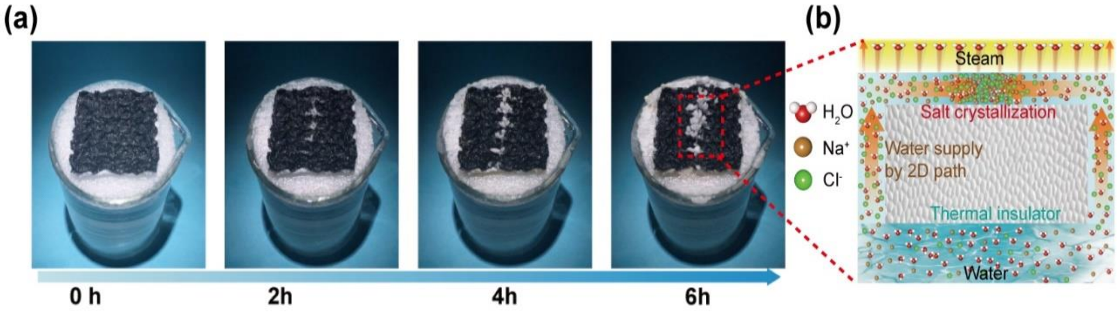

Fig. S24 a The hydrophilic honeycomb fabric is wrapped around the surface of the insulation foam to form a 2D plane water transport path, and salt accumulates on the surface of the fabric during prolonged evaporation. **b** Schematic illustrations of the transport of water and salt in the solar steam generator

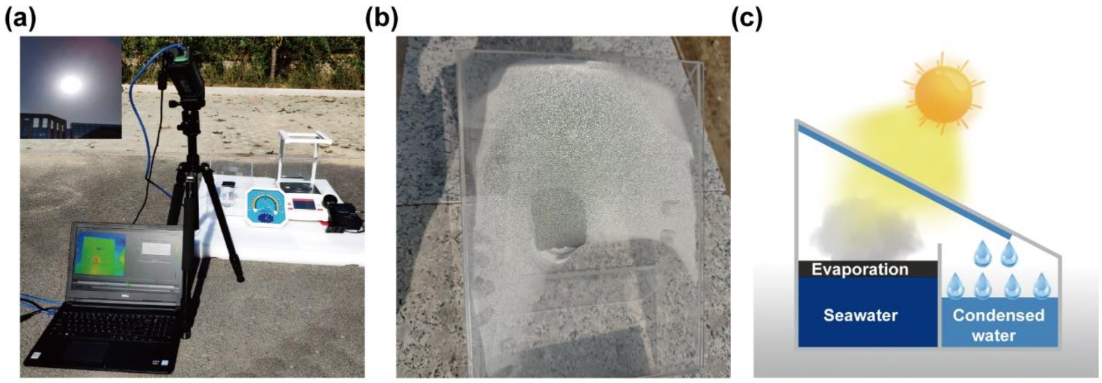

Fig. S25 a–b The actual images and **c** schematic diagram of solar distillation and condensate collection device

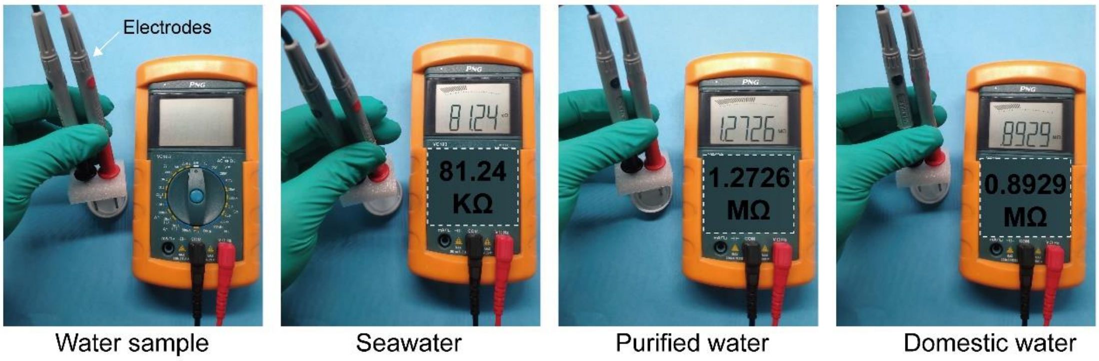

Fig. S26 The resistance of different water measured by a multimeter with constant electrode spacing

S3 Supplementary Table

Table S1 The solar-thermal performance and structure properties of materials under one sun illumination

Materials	Evaporate rate (kg m^{-2} h^{-1})	Solar-steam efficiency (%)	Refs.
CNT-coupled cotton fabrics	1.59	89.6	R1[S9]
CNT-embedded PAN nonwoven fabrics	1.44	81.0	R2[S10]
Carbon black nanoparticles of Janus absorbers	1.3	72	R3[S11]
linen fabric/CS/PU	1.447	90	R4[S12]
Carbon-coated fabrics	1.33	88.9	R5[S13]
Carbon nanotubes	1.18	86.01	R6[S14]

loading on cotton fabric			
Macroporous three-dimensional MXene	1.41	88.7	R7[S15]
Hydrophobic MXene membrane	1.31	71	R8[S16]
MXene-based sponge	1.41	84.8	R9[S17]
Janus MXene-based aerogels	1.46	87	R10[S18]
MXene/Cellulose fibrous membrane	1.44	85.8	R11[S19]
MXene-based hierarchical-foam	1.393	93.4	R12[S20]
Graphite-wood	1.15	80	R13[S21]
PPy membrane	1.12	70.3	R14[S1]
(PVDF)/graphene membranes	1.2	84	R15[S22]
Cu_2O/Cu_2S core/shell nanowires-foam	1.44	83.1	R16[S23]
3D ceramic plate	1.45	91	R17[S24]
Aluminophosphate-treated wood	1.42	90.8	R18[S25]
CNTs@SiO_2 nanofibrous aerogels	1.5	85.4	R19[S26]
**MXene-decorated 3D honeycomb-fabric**	**1.62**	**93.5**	**This work**

Supplementay References

[S1] D. Qi, Y. Liu, Y. Liu, Z. Liu, Y. Luo et al., Polymeric membranes with selective solution-diffusion for intercepting volatile organic compounds during solar-driven water remediation. Adv. Mater. **32**, 2004401 (2020). https://doi.org/10.1002/adma.202004401

[S2] H. Ghasemi, G. Ni, A.M. Marconnet, J. Loomis, S. Yerci et al., Solar steam generation by heat localization. Nat. Commun. **5**, 4449 (2014). https://doi.org/10.1038/ncomms5449

[S3] C.O. Popiel, J. Wojtkowiak, Simple formulas for thermophysical properties of liquid water for heat transfer calculations (from 0°C to 150°C). Heat Transf. Eng. **19**, 87-101 (1998). https://doi.org/10.1080/01457639808939929

[S4] P.-F. Liu, L. Miao, Z. Deng, J. Zhou, H. Su et al., A mimetic transpiration system for record high conversion efficiency in solar steam generator under

one-sun. Mater. Today Energy **8**, 166-173 (2018). https://doi.org/10.1016/j.mtener.2018.04.004

[S5] S. Chen, Z. Sun, W. Xiang, C. Shen, Z. Wang et al., Plasmonic wooden flower for highly efficient solar vapor generation. Nano Energy **76**, 104998 (2020). https://doi.org/10.1016/j.nanoen.2020.104998

[S6] Z. Liu, H. Song, D. Ji, C. Li, A. Cheney et al., Extremely cost-effective and efficient solar vapor generation under nonconcentrated illumination using thermally isolated black paper. Glob Chall. **1**, 1600003 (2017). https://doi.org/10.1002/gch2.201600003

[S7] H. Song, Y. Liu, Z. Liu, M.H. Singer, C. Li et al., Cold vapor generation beyond the input solar energy limit. Adv. Sci. **5**, 1800222 (2018). https://doi.org/10.1002/advs.201800222

[S8] W.T. Cao, F.F. Chen, Y.J. Zhu, Y.G. Zhang, Y.Y. Jiang et al., Binary strengthening and toughening of mxene/cellulose nanofiber composite paper with nacre-inspired structure and superior electromagnetic interference shielding properties. ACS Nano **12**, 4583-4593 (2018). https://doi.org/10.1021/acsnano.8b00997

[S9] H. Kou, Z. Liu, B. Zhu, D.K. Macharia, S. Ahmed et al., Recyclable CNT-coupled cotton fabrics for low-cost and efficient desalination of seawater under sunlight. Desalination **462**, 29-38 (2019). https://doi.org/10.1016/j.desal.2019.04.005

[S10] B. Zhu, H. Kou, Z. Liu, Z. Wang, D.K. Macharia et al., Flexible and washable CNT-embedded PAN nonwoven fabrics for solar-enabled evaporation and desalination of seawater. ACS Appl. Mater. Interfaces **11**, 35005-35014 (2019). https://doi.org/10.1021/acsami.9b12806

[S11] W. Xu, X. Hu, S. Zhuang, Y. Wang, X. Li et al., Flexible and salt resistant janus absorbers by electrospinning for stable and efficient solar desalination. Adv. Energy Mater. **8**, 1702884 (2018). https://doi.org/10.1002/aenm.201702884

[S12] L. Song, P. Mu, L. Geng, Q. Wang, J. Li, A novel salt-rejecting linen fabric-based solar evaporator for stable and efficient water desalination under highly saline water. ACS Sustain. Chem. Eng. **8**, 11845-11852 (2020). https://doi.org/10.1021/acssuschemeng.0c04407

[S13] Y. Li, X. Jin, Y. Zheng, W. Li, F. Zheng, W. Wang et al., Tunable water delivery in carbon-coated fabrics for high-efficiency solar vapor generation. ACS Appl. Mater. Interfaces **11**, 46938-46946 (2019). https://doi.org/10.1021/acsami.9b17360

[S14] Q. Qi, Y. Wang, W. Wang, X. Ding, D. Yu, High-efficiency solar evaporator prepared by one-step carbon nanotubes loading on cotton fabric toward water purification. Sci. Total Environ. **698**, 134136 (2020).

https://doi.org/10.1016/j.scitotenv.2019.134136

[S15] X. Zhao, X. Zha, J. Pu, L. Bai, R. Bao et al., Macroporous three-dimensional MXene architectures for highly efficient solar steam generation. J. Mater. Chem. A **7**, 10446-10455 (2019). https://doi.org/10.1039/c9ta00176j

[S16] J. Zhao, Y. Yang, C. Yang, Y. Tian, Y. Han et al., A hydrophobic surface enabled salt-blocking 2D Ti_3C_2MXene membrane for efficient and stable solar desalination. J. Mater. Chem. A **6**, 16196-16204 (2018). https://doi.org/10.1039/c8ta05569f

[S17] Q. Zhang, Z. Fu, H. Yu, S. Chen, Nanoplating of a SnO_2 thin-film on MXene-based sponge for stable and efficient solar energy conversion. J. Mater. Chem. A **8**, 8065-8074 (2020). https://doi.org/10.1039/d0ta01258k

[S18] Q. Zhang, G. Yi, Z. Fu, H. Yu, S. Chen et al., Vertically aligned Janus MXene-based aerogels for solar desalination with high efficiency and salt resistance. ACS Nano **13**, 13196-13207 (2019). https://doi.org/10.1021/acsnano.9b06180

[S19] X.J. Zha, X. Zhao, J.H. Pu, L.S. Tang, K. Ke et al., Flexible anti-biofouling MXene/cellulose fibrous membrane for sustainable solar-driven water purification. ACS Appl. Mater. Interfaces **11**, 36589-36597 (2019). https://doi.org/10.1021/acsami.9b10606

[S20] X. Fan, Y. Yang, X. Shi, Y. Liu, H. Li et al., A MXene-based hierarchical design enabling highly efficient and stable solar-water desalination with good salt resistance. Adv. Funct. Mater. 30, 2007110 (2020). https://doi.org/10.1002/adfm.202007110

[S21] T. Li, H. Liu, X. Zhao, G. Chen, J. Dai et al., Scalable and highly efficient mesoporous wood-based solar steam generation device: localized heat, rapid water transport. Adv. Funct. Mater. **28**, 1707134 (2018). https://doi.org/10.1002/adfm.201707134

[S22] C. Huang, J. Huang, Y. Chiao, C. Chang, W. Hung et al., Tailoring of a Piezo-Photo-Thermal solar evaporator for simultaneous steam and power generation. Adv. Funct. Mater. 31, 2010422 (2021). https://doi.org/10.1002/adfm.202010422

[S23] L. Ying, H. Zhu, H. Li, Z. Zhu, S. Sun et al., Heterostructure design of Cu_2O/Cu_2S core/shell nanowires for solar-driven photothermal water vaporization towards desalination. Sustain. Energy Fuels **4**, 6023-6029 (2020). https://doi.org/10.1039/d0se00914h

[S24] L. Shi, Y. Shi, S. Zhuo, C. Zhang, Y. Aldrees et al., Multi-functional 3D honeycomb ceramic plate for clean water production by heterogeneous photo-Fenton reaction and solar-driven water evaporation. Nano Energy **60**, 222-230 (2019). https://doi.org/10.1016/j.nanoen.2019.03.039

[S25] T. Chen, Z. Wu, Z. Liu, J.T. Aladejana, X.A. Wang et al., Hierarchical porous

aluminophosphate-treated wood for high-efficiency solar steam generation. ACS Appl. Mater. Interfaces **12**, 19511-19518 (2020). https://doi.org/10.1021/acsami.0c01815

[S26] X. Dong, L. Cao, Y. Si, B. Ding, H. Deng, Cellular structured CNTs@SiO2 nanofibrous aerogels with vertically aligned vessels for salt-resistant solar desalination. Adv. Mater. **32**, 1908269 (2020). https://doi.org/10.1002/adma.201908269
